# 

**Published:** 1902-10-04

**Authors:** 


### The Hospital

A JOURNAL OF

THE MEDICAL SCIENCES AND HOSPITAL ADMINISTRATION,

INCLUDING

### A SPECIAL SECTION FOR NURSES.

IN TWO VOLUMES ANNUALLY.

LIBRARY

SURGEON GENERAL'S OFFICE

NOV. 2--1903

18285-0.

EDITED BY

SIR HENRY BURDETT, K.C.B.,

AND

SOLOMON C. SMITH, M.D.

VOLUME XXXIII.

OCTOBER 1902 TO MARCH 1903.

London:

PUBLISHED BY THE SCIENTIFIC PRESS (LIMITED), AT THE HOSPITAL BUILDING,
28 & 29 SOUTHAMPTON STREET, STRAND, W.C.

MDCCCCIII.

W1
HO69X

### INDEX TO VOL. XXXIII., 1903.

*** Articles under the general heading *Hospital Clinics and Medical Progress* are distinguished by the initial (C.) for *Hospital Clinics*, and by (P.) for the rest of the series. (W.) distinguishes the *Institutional Workshop* articles, and (A.) the *Annotations*.

Abandoned Children (A.), 173
Abbreviations (C.), 47
Abdomen, Diseases of the (P.), 82, 96, 115, 145, 198, 217, 231
Abdomen, Tumours of the (C.), 441
Abdominal Surgery (P.), 322
Abscess, Cerebral (P.), 15
Acute and Chronic Disease, The Relation between (C.), 177
Addenbrooke's Hospital, Cambridge (W.), 257, 344
Adenoma, Malignant, of the Kidney (C.), 94
Adenoids and Trachoma (C.), 301
Æsthetic Surgery (C.), 212
African Lethargy (P.), 391
After-care Association for the Insane (W.), 54, 113
Albumosuria, Myelopathic (P.), 409
Alcohol in Diet, 347
Alcohol in Medicine (C.), 141
Alcohol in Surgery (C.), 141
Alcoholic Parents, The Children of (A.), 191
Aliens, Education and Trachoma (A.), 155
Alkaptonuria (P.), 409
Ambulance Litter, A Light (W.), 276
Anæmia, The Œdema of (P.), 180
Anæmia, Splenic (P.), 81, Pernicious (P.), 95
Aneurysm, Aortic Inequality of the Pupils in (P.), 216
Aneurysms, Death in Unruptured (P.), 269
Ankylostomiasis (C.), 265
Anti-Scarlatinal Serum (A.), 3
Antiseptics in Chronic Dyspepsia (C.), 194
Appendicitis (P.), 320, (C.), 388
Appendicitis and the New English Dictionary (A.), 349
Appendicitis, Sub-phrenic Abscess in (C.), 337
Appendicitis, When to Operate in (C.), 282
Archbishop Designate and the League of Mercy, 328
Arsenic, The Place of, in the Production of Disease (C.), 77
Arthritis, Infective, Mr. Howard Marsh on (C.), 195
"Aseptic" and "Antiseptic" Surgery, 190
Aseptic Surgery, "So called" (C.), 335
Aspirin in the Treatment of Glycosuria and Diabetes Mellitus (C.), 265
Asthma: the Nasal Treatment (C.), 62
Asylum Holocaust, The (W.), 308
Auchterhouse, Dundee, Sanatorium for Consumptives (W.), 412
Auto-intoxication (C.), 319

Baby Farming (A.), 279
Bacillus Coli, Infection by (P.), 178
Bacteria and Butter-Making (C.), 443
Bacteria, Intestinal (C.), 247
Bacteria in the Intestine, Growth of (P.), 428
Bacterial Toxins, 419
Bacteriological Diagnosis, Uncertainties of (C.), 13
Bacteriology, Progress in (P.), 80, 178, 284, 427
Banti's Disease (P.), 179
Barber's Chair, The (A.), 191
Barnardo, Dr., and his Work (W.), 309
Beneficent Diseases (A.), 75
Beriberi (P.), 197
Bill relating to Officers of Health, The (W.), 431
Bilocular or Hour-Glass Stomach (P.), 303
Biological Test for Blood, The (P.), 162
Birth Paralysis of the Upper Extremity (C.), 336
Black Smoke, 101
Blackwater Fever (P.), 79
Bladder, Complete Excision of the (P.), 373
Bladder, Hernia of the (P.), 114
Bladder, Partial Resection of the, for Carcinoma (P.), 32
Bladder, Rupture of the (P.), 32
Bladder and Prostate, Removal of, for Carcinoma (P.), 428
Blood Counting (P.), 162
Blood, Diseases of the, Progress in (P.), 81, 95, 162, 179
Blood, Human, Bactericidal Effects of (P.), 178
Blood, Human, Trypanosome in (P.), 178
Blood Pressure, The Rise of in Later Life (C.), 301
Bookworld (*Books, Magazines and Pamphlets Reviewed*)
Beddard (and others) Practical Physiology, 283
Birmingham. *See Macnaughton-Jones*
Blumfield. Anæsthetics, A Practical Handbook, 164
Carless. *See Rose*
Carr, Pick, Doran and Duncan. Practitioner's Guide, 84
Cattell. International Clinics, 116
Chapin. Theory and Practice of Infant Feeding, 181
Chaplin. *See Colbeck*
Colbeck and Chaplin Science and Art of Prescribing, 199
Donald. The London Manual for 1903, 324
Doran. *See Carr*
Duncan. *See Carr*
Edkins. *See Beddard.*
Edwardes. Concise History of Small-pox and Vaccination in Europe, 164
Fox. Practical Treatise on Small-pox, 33
Garrigues. Science and Art of Obstetrics, 306
Haultain. Practical Handbook of Midwifery, 324
Hill. *See Beddard.*
Ikin and Lyster. Advanced Hygiene, 234
Joyce. *See Macnaughton-Jones.*
Kipping and Perkin. Organic Chemistry, 199
Latham and West. Prize Essay on a Sanatorium for Tuberculosis, 358
Lendon. Hydatid Diseases of the Lungs, 116
Lyster. *See Ikin*
Macleod. *See Beddard*
Macnaughton-Jones. Points of Practical Interest in Gynæcology, 324
Macnaughton-Jones (and others). Practitioners' Handbook of Diseases of the Ear and Nasopharynx, 18
Metropolitan Asylums Board. Annual Report, 1901, 306
Miles. Avenues to Health, 288
Milligan. *See Macnaughton-Jones*
Moor. Suggested Standards of Purity for Food and Drugs, 164
Murrell. Aids to Forensic Medicine and Toxicology, 376
Newbolt (and others). Reports of the Royal Southern Hospital, Liverpool, 306
Ostrom, Massage and the Original Swedish Movements, 199
Pembery. *See Beddard*
Perkin. *See Kipping*
Pick. *See Carr*
Psychical Research Society, Proceedings of, 84
Pyle (edited by), Manual of Personal Hygiene, 376
Rose and Carless, Manual of Surgery, 100
Saundby, Medical Ethics, 18
Schafer, Essentials of Histology, 36
Startin, the Care of the Skin and Hair, 181
Stephens, Digest of Public Health Cases, 36
Stewart, *see Macnoughton-Jones*
Tilley, *see Macnaughton-Jones*
Underwood, Aids to Dental Anatomy and Physiology, 164
Wallace, Small-pox, 116
Walsh, Age and Old Age, 234
West, *see Latham*
Whitla, Elements of Pharmacy, 324
Year Book of Scientific and Learned Societies, 234
Yeo, Manual of Medical Treatment, 100
Boy and Girl Clerks, 131
Bradshaw Lecture, The (C.), 111
Brain Abscess, Otitic (P.), 248
Brain and Nerves, Surgery of the (P.), 15
Breast, Diseases of the (C.), 423
Breeding of the Human, The (A.), 43

C.E.T.S., The and Women Drunkards, 182
Caisson Disease (P.), 216
Calcaneum, Fracture of the Greater Process of the (C.), 246
Cancer of the Breast treated by Removal of the Ovaries (C.), 30
Cancer of the Breasts and Ovary (C.), 63
Cancer of the Larynx, Early Treatment of (C.), 159
Cancer, the Parisitic Origin of (A.), 403
Cancer and the Quakers (C.), 79
Cancer of the Tongue, Early Diagnosis in (C.), 352
Cancer of the Uterus treated by "Rays" (C.), 265
Canteen in the Navy, The, 235
Cataract (C.), 447
Cataract Extraction in India (P.), 127
Cataract, Senile, Spontaneous Cure (P.), 113
Catheter, The, in Abdomino-Pelvic Operations in Women (C.), 247
Catheterisation of the Ureters and Radiography (C.), 33
Cerebral Compression (P.), 375
Certification in Lunacy, 138
Chloroma (P.), 34, 163
Cholecystectomy (P.), 391
Cholelithiasis (P.), 390
Claudication, Intermittent (P.), 216
Cocainisation in Obstetrics (C.), 424
Colney Hatch, A Lesson from (A.), 315
Commissariat, The (W.), 187
Consumption Cases, Selection of, for Sanatorium Treatment, 5
Consumption and Charity (A.), 385
Consumption in the Navy, 42
Contract Medical Practice (C.), 29; Does it Pay? (C.), 29
Convalescent Homes in London Suburbs (A.), 139
Convulsions, Non-epileptic in Growing Children (C.), 335
Co-operative Practice (A.), 243
Crèche, The, and the School, 19
Current from the Main (C.), 77
Cystoscopy, Suprapubic (P.), 32
Cysts, Tubo-Ovarian (P.), 426

Darwin, The Ill-health of (A.), 403
De-Hæmoglobinised Blood Films, The Preparation of (C.), 353
Dermatitis Herpetiformis (P.), 446
Dermatological Mystery Solved, A (C.), 143
Diarrhœa, The Seasonal Incidence of, and the Part Played by Flies (C.), 299, 317
Digestion, The Phenomena of, and their Significance (C.), 405
Digestion, Some Points in the Pathology and Therapeutics of (C.), 387
Digitalis in Heart Disease (P.), 269
Dilatation, Atonic, of the Stomach (C.), 157
Dilated Stomach, Surgical Treatment of (C.), 175, 193
Diphtheria (P.), 80
Diphtheria Bacillus, Persistence of (C.), 13
Diphtheria Bacillus and Scarlet Fever, The (P.), 285
Doctors and Education Committees, 242
Drains, The Testing of (W.), 238
Draughts v. Fresh Air (A.), 59
"Drayton Grange" The, and the "Oswestry Grange" (A.), 27
Drugs, Unexpected Results from taking (C.), 63
Drunk or Dying Cases (W.), 327; *see pp.* 363, 380, 398, 417
Dysentery (P.), 215

Ear Diseases, Chronic Non-suppurative (P.), 128
Eclampsia (C.), 389
Editor's Letter Box, 23, 39, 87, 135, 152, 168, 205, 240, 258, 294, 363, 398, 417, 435
Eighteenth-Century Practitioner of Medicine, The (A.), 439
Electricity, Recent Applications of (C.), 93, 109, 125
Empyema in Childhood (C.), 11
Empyema, The Etiology of (C.), 263
Endocarditis, Acute Rheumatic, with Congenital Heart Lesion (P.), 64
Endocarditis, Malignant (P.), 269
Enterectomy (P.), 355
Enteroptosis (C.), 21 (P.) 354
Enuresis in Childhood (C.), 211
Eosinophilia (P.), 96
Epilepsy (P.), 267
Epsom College (A.), 385
Eructations, Inflammable (C.), 64

Fever, Continuous, due to the bacillus enteridis of Gaertner (P.), 34
Fevers, Progress in (P.), 33, 266, 286, 302
Fire Extinguisher, A Novel (W.), 361
Fish, Leprosy and Mr. Hutchinson (A.), 297
Flies, The Part played by, in Diarrhœa (C.), 299
Fogs and Gas Poisoning (C.), 61
Food, The Effect of Variations in, on the Digestive Glands (C.) 369
Fourth Disease (P.), 287

Gall Bladder and Bile Ducts, The Surgery of (C.), 406
Gastrectomy (P.), 303

Gastric Contents, Examination of the (P.), 64
Gastric Ulcer (P.), 303
Gastric Vertigo (P.), 392
Gastro-Enterostomy (P.), 285
Gastrostomy (P.), 285
General Medical Council, The, 153
Genito-Urinary Surgery (P.), 16, 32, 340, 410, 428
Glances at the Hospitals (W.), 38, 54, 71, 103, 203
Gloves in Operatic Surgery (O.), 213
Goitre, Exophthalmic (C.), 95, (P.), 392
Gout (P.), 304, 321
Gowers, Sir W. B., on Syphilitic Diseases of the Nervous System (C), 142
Grave Scandal (A.), 331
Gynæcology, The Limits of (A.), 279
Gynæcology, Phototherapeutics in (P.), 410
Gynæcology, Progress in (P.), 409, 426

Hæmatemesis, Post-operative (P.), 323
Hæmophilia (P.), 96
Hæmoptysis, The Gelatin Treatment of (C.), 359
Hæmorrhage, Intraperitoneal Incident to Ectopic Gestation (P.), 426
Harveian Oration, The, 57
Health Aspects of London Locomotion (A.), 367
Health Resorts (W.), 53, 118, 279
Heart and Circulation, Diseases of the (P.), 216, 250, 268
Heart, a Gunshot Wound of the (C.), 247
Heart, Manual Compression of the in Syncope due to Anæsthetics (O.), 213
Heart, State of the, after Death from Asphyxia (P.), 196
Heart, Suturing a Wounded (C.), 247
Heat Stroke (O.), 442
Hemianopsia (C.), 443
Hemiplegias (C.), 351, 370
Hepatic Abscess, The Treatment of (C.), 176
Heredity of Mental Disease, The (P.), 339, 373
Heredity and the Question of Forced Labour (C.), 406
Hernia (P.), 97, 114
Hernia, Femoral, Inguinal, Umbilical (P.), 98, 99
Hernia, Obturator, Strangulated (P.), 114
Hint to Specialists, A (A.), 107
Hodgkin's Disease (P.), 179
Hospital Abuse in Bradford (A.), 297; 329
Hospital Administration (W.), 237, 252, 270, 291, 326, 342, 360, 377, 394, 430, 448
Hospital Expenditure (W.), 87
Hospital Meetings, 167, 185, 328, 379, 397, 414, 432, 449
Hospital Officers' Association (W.), 133
Hospital Questions which Press (W.), 327
Hospital Support: Individual and Collective Efforts, 121
Hospitals and the Aristocracy of the Poor (W.), 291
Hospitals, The Migration of, 437
Hospitals, The Unequal Distribution of, in London (W.), 394
Housing Question, The, 366
Hull Sanatorium (W.), 37
Human and Bovine Tuberculosis (A.), 315
Hydrosalpinx, Etiology of (P.), 426
Hygiene in the Barber's Shop (A.), 315
Hypnotic Treatment of Criminals, The (A.), 333
Hypnotism (A.), 385
Hysterectomy, Reaction against (O.), 45
Hysterical Mutism (P.), 375

Icthyol in Trachoma (P.), 446
Imperial Vaccination League, The (A.), 261
Inebriate Homes, 224
Infantile Mortality in Liverpool (A.), 155
Infants, The Feeding of (P.), 34
Infants, The Preservation of (A.), 107
Infection, The Sowing of (A.), 27
Influenza (P.), 33
Insane, The Story of the, from Year to Year (W.), 39, 55, 87, 203, 221, 239, 329, 345, 362
Institutional Notes and Queries (W.), 56
Insurance and Crime, 325
Intemperance among Women, 106
Internal Saphenous Vein, Ligature of (C.), 407
Intestinal Obstruction (P.), 355
Intestinal Suture (P.), 254
Intestines, Progress in Surgery of the (P.), 354
Introductories, The, 25
Intussusception (O.), 353

Jugular Vein, Ablation of the (C.), 11

Kernig's Sign (P.), 391
Kidney, Diseases of the (P.), 393, 408
Kidney, Granular (P.), 393
Kidney, Stone in the (P.), 373
Kidney, Tubercular (P.), 371
King Edward's Hospital Fund (W.), 147, 377
King's Hospital Fund, The, 137
Knee Jerk in Early Tabes (C.), 78
"Knife and Fork" Surgery (A.), 349
"Knowing" and "Looking," 41

Landry's Paralysis (P.), 392
Laryngotomy as a Preliminary to Mouth and Throat Operations (C.), 263
Law relating to Infectious Diseases, The (W.), 20
Law relating to Lunatics and Persons of Unsound Mind, The (W.), 310, 360, 488
Law affecting Medical Practitioners, The (W.), 132, 166, 219
League of Mercy, The, 135, 202
Leprosy (P.), 14
Less than Justice (A.), 139
Leucocytosis (P.), 96
Leucocytosis, Post-operative Non-septic (C.), 31
Leukæmia (P.), 82
Ligaments, Crucial, The Repair of by Open Operation (C.), 176
Light and Heat as Causes of Cataract (C.), 318
Liver and Gall Bladder, Surgery of the (P.), 390
Liver, Tropical Abscess of the (P.), 390
Liverpool Hospital for Consumptives at Delamere Forest (W.), 183
Liverpool Infirmary for Sick Children (W.), 21
London Ambulance Question, The (A.), 191
London Ambulance Service, The (W.), 200
London County Council, The, and Overcrowding (A.), 123
London County Council, The, and Tottenham Hospital, 105
London Health Report, The, 259
London Hospital, The (W.), 272, (A.), 403
London Hospital Appeal, The (A.), 279
London Hospital, Alterations at the (W.), 185
London Life Table, The, 89
London Locomotion, 402
London's New Pathologist (A.), 225
London's New Pay Hospital (W.), 273
Long, Mr., and Vaccination, 277
Loose Prescribing, 365
Lorenz, Professor, and his Method (A.), 261
Lunacy Act, Proposed Amendments to the (C.), 281, 299
Lunacy, The Hospital Treatment of 74
Lunatic Asylums in the Bombay Presidency, the North-Western Province and Oudh (W.), 102
Lyon-Cadette Method of Ventilation, The (W.), 292

Madras Presidency, The Civil Hospitals and Dispensaries of the (W.), 220
Malaria (P.), 79
"Malt" and Other Whiskies (A.) 297
Manchester Infirmary, The (A.), 59
Manchester Infirmary, Rejuvenescence of the (A.), 91
Mankind in the Marring (A.), 243
Mansion House and the Hospitals, The, 58
Meckel's Diverticulum, Hernia of (P.), 115
Medical Acts, Amendment of the, 189
Medical Department of the Royal Navy, The (W.), 68
Medical Education, Preliminary, 171
Medical Inspection of Schools, The, 332
Medicine, Orthodox and Unorthodox, 26
Medicine Stamp Question, The (W.), 37
Membrana Tympani, Perforation of the, by Lightning Stroke (P.), 129
Mercury in Perforating Wounds of the Globe (P.), 446
Metropolitan Asylums Board, The (A.), 123
Metropolitan Hospital Sunday Fund, The (W.), 184, 257
Micrococcus of Acute Rheumatism, The (C.), 337
Middle-Ear Disease (P.), 128
Middlesex Hospital, The (W.), 168
Minor Ailments, The Pathology of (A.), 91
Modern Sociology, 19, 52, 101, 131, 183, 218, 235, 289, 307, 325, 359
More Schemes (A.), 439
Morphia Habit (P.), 49
Morphinism (P.), 49
Mosquito, The Natural Enemies of the (C.), 12
Motor Aphasia from Injury (P.), 375
Municipalisation of all Hospitals, The (W.), 165
Municipalisation or Moonshine? (W.), 150
Myocardium (P.), 250

Nephrectomy, Partial (P.), 17
Nerve Degeneration, Pathology of (P.), 391
Neurasthenia, Post-operative (A.), 75
Neurology, Progress in (P.), 249, 267, 374, 391
New Appliances and Things Medical, 17, 35, 51, 67, 83, 99, 130, 180, 251, 305, 323, 341, 357, 393, 411, 429, 447
1902 (O.), 227
Nordrach Sanatorium (W.), 37
Notes and News, 4, 28, 44, 60, 76, 92, 108, 124, 140, 156, 174, 192, 210, 226, 245, 262, 280, 298, 316, 334, 350, 368, 386, 404, 422, 440
Notification of Measles, The, 278
Nottinghamshire Sanatorium for Consumptives (W.), 343. *See p.* 380

Odours of Disease, The (A.), 89
Œdema, Acute, of the Eyelids in Children (P.), 50
Œdema, Persistent Hereditary, of the Lower Limbs (P.), 196
Old Age, The Study of (A.), 59
Operation, The Limits of an (A.), 196
Operation Mania (A.), 43
Ophthalmia, Infantile (P.), 113
Ophthalmia, Phlyctenular (C.), 282
Ophthalmology, Progress in (P.), 113, 127, 446
Orthodoxy and Dissent, 295
Otitis Media, Suppurative (P.), 232
Otology (P.), 232, 248
Ovary, Fibromata of the (P.), 427

Pancreas and Spleen, Progress in Surgery of the (P.), 355
Pancreatitis, Chronic (P.), 355
Papilloma, Villous, of the Renal Pelvis (P.), 341
Paraffin Noses (O.), 282
Paralyses of Children, Surgery of (C.), 370
Paralysis, Functional and Organic, Diagnosis of (P.), 249
Paralysis, General (P.), 65
Paralysis, Spastic Spinal (P.), 267
Pathological Society, The (C.), 158
Pawnshop Pathology of the (A.), 107
Pay Hospital for London, A (W.), 255
Pediatrics, Progress in (P.), 34, 50, 64
Perforation in Typhoid Fever, Operation for (C.), 283
Pericardium, Adherent (C.), 443
Peritonitis (P.), 322
Peritonitis, Pneumococcal (C.), 214
Phototherapy, Progress in (P.), 129, 146, 160
Plague (P.), 427
Plague, The History of, 90. *See p.* 135
Plague in San Francisco (A.), 173
Pleasure as a Vaso-dilator (C.), 61
Pneumococcus (P.), 285
Pneumonia (C.), 126
Pneumonia, Hospital (A.), 375
Poore's Dr. Vivian, Views (C.), 47
Poverty, The Advantages of, 438
Practical Department (W.), 188, 276, 344, 351, 380
Practitioner and the Public, The (A.), 421
Pregnancy, The Heart in (P.), 269
Prize Plans for the King's Sanatorium, The, 241
Propagation of the Unfit (A.), 173
Prostate, Enlarged, Operative Treatment of the (P.), 411
Prostate, Senile Enlargement of the (P.), 410
Prostate, Tubercular Disease of the (P.), 428
Psychiatry, Progress in (P.), 49, 65, 339, 373
Public House, The, and the Spread of Consumption (A.), 91
Puerperal Aphasia (P.), 375
Puerperal Fever, The Treatment of, by Formalin (C), 352
Pyelitis, Acute, in Infancy (P.), 35
Pylorus, Malignant Disease of the (C.), 143

Question for Boards of Guardians, A (W.), 291

Race and Mortality, 172

Radiotherapy, Progress in (P.), 338, 356

Raw Meat in Pulmonary Tuberculosis (P.), 50

Recklinghausen's Disease (P.), 446

Registrar-General's Return, The, 122

Renal Sufficiency, Determination of (P.), 373

Restaurant Kitchens (A.), 123

Retina, Detachment of the (P.), 127

Revolt in the Kindergarten, The (A.), 439

Rheumatic Fever (P.), 427

Rheumatic Fever, Surgery in (C.), 320

Rheumatism Acute (P.), 305

Rheumatism, Dilatation of the Heart in (P.), 268

Rheumatism and Gout (P.), 304, 321

Roentgen Ray Treatment of Rodent Ulcer (O.), 245

Roentgen Ray Work with the Alternating Current (W.), 251

Rural Education, 313

St. Bartholomew's, The Crisis of (A.), 357
St. Bartholomew's Committee of Inquiry (W.), 396
St. Bartholomew's Hospital (W.), 290, 383, 435
St. Bartholomew's Hospital, The Future of (W.), 270
St. George's Hospital (W.), 236
Salvation Army, The Social Work of the, 52
Sanitary Expenditure and Local Indebtedness, 260
Sanitary Measures and Popular Susceptibilities (A.), 27
Scarlet Fever (P.), 302
Scarlet Fever, Period of Infectivity in (A.), 349
Schools and Sanitary Authorities (W.), 219
Sclerosis of the Superficial Veins (P.), 216
Sclerotic, Rupture of the (P.), 113
Scurvy, Infantile (P.), 65
Sealing Wounds, A New Way of (C.), 283
Seborrhœa (P.), 447
Serviette Question in Open-Air Sanatoria (W.), 344
Sevenoaks, Opening of a New Hospital at (W.), 69
Sinus Thrombosis, Infective Lateral (P.), 248
"Sitting on the Pill" (A.), 269
Skin Diseases, Progress in (P.), 14, 446
Sleeping Sickness in Uganda, 207
Slum Experiment A, 307
Small-Pox at Purfleet, 334
Small-Pox and Vaccination (P.), 43
Snarl from Birmingham, A (A.), 3
Soldiers' Deaths in Peace Time (A.), 155
Spasmus Nutans (P.), 51
Spastic and Syphilitic Spinal Paralysis (C.), 46
Spina Bifida (P.), 16

Spinal Disease, Gastric Symptoms in (O.), 300
Spine, Forcible Straightening of the, in Paraplegia (O.), 212
Splanchnoptosis (P.), 354
Spleen, Rupture of the (P.), 356
Spondylitis Deformans (P.), 321
Sporadic Typhoid, 314
Staphylococcus Vaccine (P.), 15
State Medicine, Progress in (P.), 144, 161, 230, 444
State and Schools (P.) 230
State-aided Hospitals, If we had (W.), 430
Stenosis Congenital, of the Aorta, at the Isthmus (P.), 269
Sterilising Instruments, A Handy Method of (O.), 159
Stomach, Progress in Surgery of the (P.), 285, 303
Student of Medicine, The, 24, 40, 56, 72, 88, 104, 120, 169, 188, 206, 222, 240, 276, 294, 312, 330, 346, 264, 381, 400, 417, 436, 454
Summer Diarrhœa, The Cause of (O.), 14
Superstition, A Waning, 2
Surgeons in the Days of Elizabeth (A.), 333
Surgery, Abdominal (P.), 322
Surgery of the Brain and Nerves (P.), 15
Surgery of the Gall-Bladder and Bile Ducts (O.) 406
Surgery, Genito-urinary (P.), 16, 32, 340, 410, 428
Surgery of the Intestines (P.), 354
Surgery of the Liver and Gall Bladder (P.), 390
Surgery of the Pancreas and Spleen (P.), 355
Surgery, Progress in (P.), 16, 32, 97, 114, 128, 232, 248, 340, 371
Surgery of the Stomach (P.), 285, 303
Surgery of the Ureters (P.), 372
Surgery of the Vermiform Appendix (P.), 320
Surgical Aid Society, The, 186
Synovitis, Gonorrhœal, Treatment of (O.), 265
Syphilis and Insanity (P.), 374
Syphilis, Tuberculosis, and Fresh Air (O.), 388

Tabes (P.), 249
Task of the new Water Authority, The, 238
Taxis, The Dangers of (O.), 176
Temperature of Workrooms, The, 296
Tetanus (P.), 33
Tetanus Infection in Gelatine (A.), 243
Theatre Fires and their Prevention (W.), 239
Thoughts on Methods of Charity, 289
Thrombosis after Typhoid Fever (O.), 158
Thrombosis Venous in Heart Disease (P.), 196
"To Stay at Home is Best" (A.), 421
Tottenham Hospital Bazaar (W.), 85
Trail of the Skirt, The (A.), 107
Treatment by Mechanical Vibration, 348
Tropical Diseases, Progress in (P.), 197, 215
Tubercular Kidney (P.) 16
Tuberculosis (P.), 408, 444
Tuberculosis, Congenital (O.), 281
Tuberculosis, The Congress on, 73
Tuberculosis, The Extinction of (O.), 425
Tuberculosis, Immunity to, possessed by Healthy Men, 264
Tuberculosis, The Prevention of, 420
Tuberculous Soil, The (A.), 333
"Tubes" *versus* "Points" (A.), 209
Tumour, Post-rectal Dermoid, containing True Bone (P.), 64
Tumours of the Bladder, Treatment of (O.), 217
Tumours of the Mesentery (P.), 354
Two Ways of looking at an Oyster (A.), 225
Typhoid Bacilli and the Soil (P.), 80
Typhoid Bacillus, The, 1
Typhoid Excreta, Treatment of (O.), 43
Typhoid Fever (P.), 67, 266, 286
Typhoid Fever Infection (A.), 261
Typhoid Fever, Prevention of, in Armies (O.), 94
Typhoid Psychosis (P.), 392

Ulceration of the Tongue, Traumatic (O.), 31

Unanimity, The Power of, 154

Underclothing, The Sterilisation of (O.), 194

Universities, The Multiplication of (A.), 209

University of London Election, The (A.), 225

Uræmia (P.), 408

Ureter, Grafting the Divided (P.), 32

Ureters severed during Abdominal Operations, Treatment of (P.), 322

Uterine Fibroids, Choice of Operations for (O.), 45

Uterus, Fibroma of the, in a Child of 13 (P.), 410

Uterus, Inversion of the (O.), 62

Uterus, Malignant Disease of the (P.), 409

Uterus, Retroflexion of the (P.), 426

Vaccination Act, The, 401
Vaginal Route for Operation, The (P.), 426
Venesection (P.), 269
Venesection in Acute Nephritis (P.), 408
Venous Dystrophy (P.), 50
Vermiform Appendix, Hernia and Strangulation of the (P.), 114
Vermiform Appendix, Surgery of the (P.), 320
Visits to Private Asylums (W.), 201, 220, 258, 293, 413
Voluntary Hospitals, The (W.), 343

Wage Earners and Sickness, 223
Wellingborough Cottage Hospital (W.), 316
Where our Loaves are made, 359
Word-building by Scientists (A.), 43
"Writing on the Wall," The (A.), 139

X-Rays (P.), 129

X-Rays in Cancer, The (O.), 177

X-Rays in Sarcoma, The (O.), 30

Zinc Gelatine, The Use of (O.), 31

Zola, The Death of (A.), 3

---

### THE SPECIAL SECTION FOR NURSES.

Addenbrooke's Hospital, Cambridge, Fire at, 30
After Fifty: or Poor Law Nurses and Pensions, 263, 277, 303
Appointments, 18, 34, 47, 60, 72, 84, 97, 111, 122, 135, 161, 174, 184, 200, 214, 226, 242, 253, 267, 280, 291, 304, 319, 330, 348, 360
Army Nursing, Past and Future, 59
Asylum Nurse, The, 292

Beyond the Seas: The Lepers' Home in Jamaica, 14

Bishop of London on Nursing, The, 265

Book and its Story, A, 49, 113, 186, 223, 269, 321

British Gynæcological Society, The, 358

British Nurses' Association and the Bona Fide Nurse, The, 254

Brooklyn Institute for Nurses and Nursing, The, 317

Camera, With a, in Hospital, 7
Charing Cross Hospital: The Nurses' Home, 252
Charing Cross Hospital: Resignation of the Lady Superintendent, 55
Christmas Books, 159, 170
Christmas Distribution, Our, 193
Christmas in the Hospitals, 195, 211
Christmas in North Germany, 181
Christmas Presents, 160
Christmas in the Workhouse Infirmaries, 144
Civil Hospitals, The, and the Army Nursing Service Reserve, 199, 210
County Nursing Associations Affiliated to Queen Victoria's Jubilee Institute, The, 72

Day in a German Klinik, A, 239

Day's Work in a Boer Refugee Camp, A, 133

Death in Our Ranks, 97, 226, 254, 267, 281, 304, 319, 348

East London Nurses: Annual Meeting, 331

Echoes from the Outside World, 22, 35, 48, 62, 75, 86, 99, 112, 124, 139, 175, 185, 201, 215, 227, 243, 255, 268, 282, 295, 307, 320, 332, 349, 361

English Nordrach, An, 15

Everybody's Opinion, 19, 32, 46, 60, 73, 83, 96, 109, 121, 136, 173, 200, 213, 225, 241, 253, 266, 279, 291, 305, 318, 330, 347, 360

Examination Questions for Nurses, 20, 70, 120, 183, 251, 316

Fracture of the Leg, A Case of Simple, 120

Fractured Pelvis, Precautions in the Case of, 252

German Society of the Red Cross; Meeting at Elberfeld, 105
Guy's Hospital: Opening of the New Ward, 359

Heaven-born Patient, The, 11

Help the Nurses to Help the Sick, 143

THE HOSPITAL Convalescent Fund, 135, 161, 174, 200, 214, 222, 242, 281, 305, 348

Isolation of Infectious cases in a Private House, The, 183

Kensington District Nursing Association, 315

League of Mercy Meeting at Kensington, 337

Lectures to Nurses on Anatomy, 4, 40, 68, 92

Lectures to Nurses on Clinical Chemistry, 118, 166, 194, 222, 248

Lectures to Nurses on Medical Nursing, 28, 54, 80, 104, 130, 180

Lectures to Nurses on Medical and Surgical Nursing, 276, 302, 328, 356

Lectures to Nurses on Ophthalmic Nursing, 208, 236, 262, 290, 314, 340

Londonderry, Lady, on District Nursing, 359

Midwives Act, The, 143

Night in the Male Surgical Ward, A, 29
Notes on News from the Nursing World, 1, 25, 37, 51, 65, 77, 89, 101, 115, 127, 141, 163, 176, 189, 204, 218, 231, 245, 257, 271, 285, 297, 310, 323, 335, 351
Novelties for Nurses, 21, 85, 169, 267
Nurses' Bookshelf, 21, 254, 306, 347
Nurses of the Central London Sick Asylum, Hendon, 249
Nurses' Co-operation, The, 55, 305
Nurses of the Hospital for Sick Children, 172
Nurses of Kensington Infirmary, The, 119
Nurse's Life in a School, A, 134
Nurses' New Quarters at Woolwich: Opening by the Queen, 291
Nurses of the Northampton General Infirmary, 57
Nurse's Story, The, 106
Nursing in the Island of Jersey, 5
Nursing by Orderlies, 108
Nursing Outlook, The, 193, 207, 221, 235, 261, 275, 289, 301, 313, 327, 339, 355
Nursing Small-Pox in a Lancashire Hospital, 111
Nursing in Smyrna, 223

Plasmon Demonstration, A, 214
Preparations for an Abdominal Operation in a Private House, 316
Presentation to Miss Annie Hobbs, 98
Presentations, 18, 30, 60, 71, 85, 97, 110, 138, 161, 169, 200, 214, 254, 281, 306, 331

"Qualified Nurse," The, 237
Queen Alexandra's Imperial Military Nursing Service, 42
Queen Alexandra's Royal Naval Nursing Service, 131
Queen Victoria's Jubilee Institute for Nurses, 240

Royal National Pension Fund for Nurses, 341
Royal Red Cross, The, 69
Royal Southern Hospital, Liverpool, The Late Matron of the, 83

Salary Question in the Colonies, 93

Sketches of our Hospital, 8, 81

Sponging Typhoids in America, 10

State Registration of Nurses, 250, 264

Stormy Side of Burgher Camp Life, The, 41

Supporting a Patient in a Bath, 70

Travel Notes and Queries, 24, 34, 64, 74, 88, 126, 184, 183, 214, 230, 267, 284, 306, 319, 331, 334, 348

Victorian Trained Nurses' Association, The, 169

Visit to the Hospitals at Beyrout, 16

Visit to the Santa Maria Nuova Ospedale, 278

Where to Go, 85, 106, 305
Words of Advice to Nurses, 12, 31, 56, 95, 107, 224
Workhouse Nursing, Report of the Departmental Committee on, 167, 182, 209
Workhouse Nursing, Mr. Sydney Holland on, 329

Printed by SPOTTISWOODE & CO. LTD., New Street Square, E.C.; and Published by the Proprietors, THE SCIENTIFIC PRESS, LIMITED, at 28 & 29 Southampton Street, Strand, London, W.C.

**Notices of Births, Deaths, and Marriages** are inserted in this column at a charge of 2s. 6d. for an announcement not exceeding four lines.

**DEATH.**

We regret to announce the death at Shoreham on the 27th September, 1902, of Miss Florence Salway, youngest daughter of the late Mr. Thomas Salway, of Chard, late of Guy's Hospital and the Highgate Medical and Surgical Home, Oak Villas, Archway Road, after three days' illness. (160)

### NOTICE TO CORRESPONDENTS.

**All MSS., Letters, Books for Review, and other matters intended for the Editor should be addressed The Editor, "The Hospital," The Hospital Building, 28 & 29 Southampton Street, Strand, W.C.**
**The Editor cannot undertake to return rejected MS., even when accompanied by stamped directed envelope.**

**Notice to Advertisers and Subscribers.**

All Advertisements, Orders for Copies of The Hospital, and Business Communications should be addressed to **THE MANAGER, (Not to the Editor)**, The Hospital, 28 & 29 Southampton Street, Strand, London, W.C.

The Cost of Subscription, post free, is as follows.—Per week, 2½d.; per quarter, 2s. 8d.; per half-year, 5s. 3d.; per annum, 10s. 6d. Foreign:—Per annum, 15s. 2d., payable, in all cases, in advance.

**NOTICE.—Vol. XXXI. of "The Hospital" (October 1901, to March 1902) in handsome cloth binding is now on sale at the office of this Journal, price, 7s. 6d. Cases for binding the Numbers contained in this Volume 1s. 6d. Index to Vol. XXXI., 2½d., post free.**

**The Monthly Part for October is now ready. Price 6d., by post 8d.**

### Notes and News.

### BOOKS RECEIVED.

The Department of Agriculture, North-West Territories.

"The Duties of the Public in Respect to Infectious and Contagious Diseases."

Frederick Clarke, Guernsey.

"A Short History of the Victoria Cottage Hospital." By E. Laurie Robinson, M.R.C.S.

J. and A. Churchill.

"Ventilation as a Dynamical Problem." By W. N. Shaw, F.R.S.

Smith, Elder and Co.

"A Clinical Lecture on Fractures in the Neighbourhood of Joints and in Sprains and Dislocations." By E. Noble Smith, F.R.C.S.Edin.

Bennett Brothers, Bristol.

"Annual Report of the Medical Officer of Health of the City and County of Bristol, 1901."

Hudson and Son, Birmingham.

"Annual Report of the Medical Officer of Health of the City of Birmingham." By Alfred Hill, M.D., F.R.S.E.

Whitley and Booth, Halifax.

"Annual Report of the Medical Officer of Health of the County Borough of Halifax."

### EDITOR'S LETTER-BOX.

### SCRAPS AND GLEANINGS.

MR. JAMES OGDEN, of Rochdale, has presented £1,000 to the Rochdale Infirmary with which to endow a bed in memory of his mother.

THE receipts of the Coventry and Warwickshire Hospital during 1901 were £536 below the ordinary expenditure. Over £1,500 is owing on the general account and nearly £600 has to be found to meet the cost and equipment of the new isolation ward. An appeal for an additional annual income of £500 has been published, signed by the Mayor, Lady Anne Murray, and Lord Leigh, and it is hoped that this sum may be forthcoming from new subscribers. During last year the annual subscriptions amounted to only £850, while the contributions of the working men have for some years past exceeded £1,000 per annum.

THE final meeting of the executive committee of the fund raised to build a new infirmary in Newcastle-on-Tyne was held recently, when the statement of accounts was presented. This statement shows that the amount raised has been £110,453, of which £108,824 has been handed over to the Governors of the Royal Infirmary, the balance of £1,629 being for expenses in connection with raising this amount.

The Library Assembly Rooms and Cottage Homes at Newborough, the foundation stone of which has been recently laid, were presented at a cost of £12,000 by Mr. Prichard Jones, of London, who is a native of Newborough.

The Danbury Holiday Home, which was started in 1890, is greatly in need of financial support. The object of the institution is to provide a home for little Londoners where they can have pure country air, good food, and motherly care. During the twelve years of its existence only twice has any illness occurred, and that of a trifling nature.

The Hop-picking Mission is this year sending to the hop fields thirteen trained nurses and twenty-five experienced lady workers to work from fourteen centres, opening small hospitals for children, dispensaries for first aid, etc., coffee stalls and barrows, and distributing illustrated papers, magazines, etc., as well as opening reading and writing rooms in the evening and on Sundays. The Rev. J. E. Revington-Jones, Mereworth Rectory, Maidstone, will gladly receive money, nursing requisites, and literature of all kinds.

The fourteenth annual charity fête which was held at Elm Grove, Sydenham, recently, proved a great success, and it is hoped that the following institutions will benefit to the extent of nearly £20 each:—The Boys' Industrial Home, the Home for Sick Children, the Sydenham Dispensary, the Girls' Industrial Home, the Sydenham and Forest Hill Charity Society, Forest Hill Dispensary, and the Forest Hill Day Nursery.

### THE STUDENT OF MEDICINE.

### APPOINTMENTS.

**Wm. Leslie Beaton**, M.B., Ch.B.Aberd., appointed Junior House Surgeon to the West Ham and East London Hospital. **J. Booth Clarkson**, L.R.C.P., L.R.C.S.Edin., appointed District Surgeon for the District of Umzinto, Natal. **Joseph E. Dods**, M.B., appointed Government Medical Officer for Brisbane, and Health Officer, Port of Brisbane. **A. O. M. Fehrsen**, B.A.Cantab., M.R.C.S.Eng., L.R.C.P.Lond., appointed Resident Surgical Officer to the Birmingham Workhouse Infirmary. **Cooper Hardcastle**, M.B., M.S.Edin., appointed Government Medical Officer and Vaccinator at Hillgrove, New South Wales. **Reginald H. Hill**, L.S.A., appointed Government Medical Officer and Vaccinator at Tocumwell, New South Wales. **T. E. Ick**, M.B.Melb., appointed Health Officer at Broad Arrow, Western Australia. **T. J. M. Kennedy**, M.B., Ch.B.Melb., appointed Port Health Officer at Geelong, Victoria. **Cyril Lewis**, M.D., C.M.Edin., appointed Honorary Anæsthetist to the Cardiff Infirmary. **J. McDonald**, M.B., C.M., appointed District Medical Officer of the Romford Union. **John Hugh Mackenzie**, F.R.C.S., appointed Health Officer for the Shire of Bulla, Victoria. **Sidney Philip Matthews**, M.R.C.S., L.R.C.P., appointed Medical Officer and Public Vaccinator for the Fourth District of the East Grinstead Union. **J. S. J. Morrison**, M.A.Cantab., M.Sc.Birm., F.R.C.S.Eng., appointed Honorary Surgeon to the Queen's Hospital, Birmingham. **A. F. M. Powell**, M.B., C.M.Edin., appointed Medical Officer to the Isle of Thanet Union Workhouse. **William Sheen**, M.S., M.D.Lond., F.R.C.S.Eng., appointed Honorary Surgeon to the "Hamadryad," Seamen's Hospital, Cardiff. **T. P. Thomas**, B.A.Cantab., M.R.C.S.Eng., L.R.C.P., appointed Honorary Surgeon to the Aberystwith Infirmary and Cardiganshire General Hospital. **Johnstone Simon Thwaites**, M.B.Melb., appointed Health Officer for the Shire of Mansfield, Victoria. **P. M. Turnbull**, M.B., Ch.B.Aberd., appointed Third Assistant Medical Officer to Bethnal Green Infirmary, Cambridge Heath. **Francis Wm. West**, M.B., M.Ch.Syd., appointed Government Medical Officer and Vaccinator at Camden, New South Wales. **Miss Ada Wilkinson**, M.B., appointed Assistant Medical Officer to the Isle of Wight County Asylum.

### "The Hospital" Institutional Library.

"Hospitals and Asylums of the World." 4 Vols., with a Portfolio of Plans, complete £12 12s.
"Burdett's Hospitals and Charities, 1902." 5s.
"Cottage Hospitals, General, Fever, and Convalescent." 10s. 6d.
"Hospital Expenditure: The Commissariat." 2s. 6d.
"A Legal Handbook for the Use of Hospital Authorities." 2s. 6d.
"The Cottage Hospital Case Book or Register of Patients." 10s. and 12s. 6d.
"The Furnishing and Appliances of a Cottage Hospital." 6d.
All these are published by the Scientific Press, Ltd., and may be obtained through any bookseller, or direct from the publishers, 28 and 29 Southampton Street, Strand, London, W.C.

